# Influence of limited replacement of NaCl with KCl and yeast extract on microbiological, chemical, sensory, and textural properties of emulsion‐type chicken sausages

**DOI:** 10.1002/fsn3.2216

**Published:** 2021-03-03

**Authors:** Majid Mohammadzadeh, Enayat Berizi, Seyed Shahram Shekarforoush

**Affiliations:** ^1^ Nutrition Research Center Department of Food Hygiene and Quality Control School of Nutrition and Food Sciences Shiraz University of Medical Sciences Shiraz Iran; ^2^ Department of Food Hygiene and Public Health School of Veterinary Medicine Shiraz University Shiraz Iran

**Keywords:** emulsion‐type sausage, KCl replacement, reduced salt, yeast extract

## Abstract

‏In this study, the production of emulsion‐type sausage by replacement of 20% to 40% KCl plus 1% or 2% yeast extract instead of NaCl was studied. The physical, chemical, microbiological, and sensory properties of the samples were analyzed up to 28 days of storage. The sample sausages were approved by the panelists. The physical, chemical, and microbiological properties of emulsion‐type sausages were not considerably influenced by these changes. The 40% replaced salt by KCl showed a similar property rather than the regular sausages. The aroma and taste of the sausages improved by using yeast extract. In addition, the adverse flavors resulted from adding KCl were excluded by adding the yeast extract. As a result, production of healthy emulsion‐type sausages (having 40% lower NaCl) with acceptable sensory qualities was introduced.

## Graphical abstract

The production of emulsion‐type sausage by replacement of 20% to 40% of KCl plus 1% or 2% of yeast extract instead of NaCl was studied. The 40% replaced salt by KCl showed a similar property rather than the regular sausages. As a result, production of healthy emulsion‐type sausages (having 40% lower NaCl) with acceptable sensory qualities was introduced.

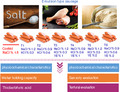

## 1 INTRODUCTION

Different factors affect human health, with food being one of the most important items. Today, with the advent of science and technology, researchers are striving to provide healthy foods to the public. Meanwhile, people are more conscious about their diet and its effect on their bodies, and they try to choose healthy foods. Meat products, especially sausages, are among the most consumed products in the world today and have a special place in stores. Nevertheless, these products contain high energy and salt content with chemical additives. To modify these products, great attempts should be made to reduce the harmful and unhealthy constituents in the formulation, and to enhance the marketing of these products. Several studies have proved the direct relationship between high sodium intake and hypertension (Ezzati et al., 2002; Gelabert et al., 2003; MacGregor, 1997; Stamler et al., 1996). Most meat products contain sodium due to the addition of salt during preparation; thus, producing low‐sodium food products is highly demanded in the meat industries (Brown et al., 2009). Approximately, 20% to 30% of the daily consumption of NaCl is associated with the consumption of meat products. The intake of different amounts of NaCl (9–12 g/day) is reported from the developed countries, which exceeds the recommended amount of 5 g/day (Jiménez‐Colmenero et al., 2001; WHO, 2003). According to the estimates in the United States alone, if salt is reduced to less than 3 g/day, the mortality rate and costs associated with treating the disease are significantly diminished (Bibbins‐Domingo et al., 2010). It is estimated that approximately 75% of sodium is consumed through materials processed in factories (Brown et al., 2009). As a response, there is a growing interest in reducing salt in foods (Wirth, 1989). Furthermore, there is a close relationship between NaCl and few textural properties in the meat products (Gou et al., 1996). Reducing salt in fermented sausages affects the technological quality, sensory properties, and microbial safety of the products (Ruusunen & Puolanne, 2005). The replacement of salt with other compounds requires them to be similar in structure to the technology and improve the sensory and microbial status of the product. One of the most important strategies to reduce the intake of NaCl is to replace it with other chloride salts, where replacing sodium chloride with potassium chloride is one of the most significant methods (Champagne et al., 1993; Gelabert et al., 2003; Guàrdia et al., 2008). Potassium chloride has a similar inhibitory effect on pathogens compared to sodium chloride, and it is generally recognized as safe (GRAS) for food supplementation (Bidlas & Lambert, 2008). However, adding high potassium chloride to meat products can cause bitter taste and reduce the salty taste in these products, which is a restrictive factor for using KCl instead of NaCl (Gelabert et al., 2003; Gimeno et al., 1998; Gou et al., 1996).

Today, using compounds to improve the taste of meat products and reduce salt intake is under extensive study. In this regard, yeast extracts (YE) are used as a natural source to enhance the good and delicious flavor in meat products (Mahadevan & Farmer, 2006). As far as the researchers investigated, no study has considered using YE and KCl to reduce sodium content in emulsion‐type sausages so far. Therefore, this study aimed to evaluate physicochemical, microbiological, and sensory properties of sausages with reduced amounts of sodium using KCl combined with YE.

## 2 MATERIALS AND METHODS

### 2.1 Treatments

In this study, the replacement of 20% and 40% NaCl (Fluka, Germany) with KCl (Merck, Darmstadt, Germany) in the emulsion‐type sausage was investigated. In addition, 1% and 2% YE (purchased from Taligene Pars Company, Isfahan, Iran) produced by *Saccharomyces cerevisiae* were added to the product (Table 1).

**TABLE 1:** Percentages of NaCl, KCl, and YE used in the emulsion‐type sausage treatments

	Treatment groups (%)						
	Control	T1	T2	T3	T4	T5	T6
NaCl	1.5	1.2	0.9	1.2	0.9	1.2	0.9
KCl	–	0.3	0.6	0.3	0.6	0.3	0.6
YE	–	–	–	1.0	1.0	2.0	2.0

### 2.2 Preparation of sausage

The sausage ingredient was prepared based on the Safir Co Meat Processing recipe, Shiraz, Iran. The basic formulation was 60% minced chicken meat, 10% vegetable oil, 18% ice flake, 5% wheat flour, 120 ppm sodium nitrate, 0.3% sodium polyphosphate, 2% gluten, sugar, ascorbic acid, sodium ascorbate, and spices. Once mixed, the appropriate amounts of NaCl, KCl, and YE were added (Table 1). Once chopped in a cutter, the dough was packed in 70 mm diameter using automatic filler cellulose casing, which was subsequently heated at 85ºC moisture compartment for 90 min. The samples were finally stored at 4°C.

### 2.3 Physicochemical analysis

The moisture (oven air‐drying method) was examined according to the instructions provided by the Association of Official Agricultural Chemists (AOAC) (Horwitz, 1975). Water activity (a_w_) was then quantified at 25°C by a Lab‐Master a_w_ (Novasina, Switzerland), and the pH was recorded on days 0, 7, 14, 21, and 28. The pH was determined in the homogenates prepared with 5 g of samples and 30 ml of distilled water (Diax 900, Heidolph) using the Metrohm 827 pH‐meter (Horwitz, 1975). Two sausages from each group were used to measure the concentration of sodium; 1 g of each homogenized sample was weighed in a pre‐acid‐washed crucible and ashed in a muffle furnace for about 5 hr at 500°C. The white ash was dissolved in 5 ml of HCI 6 M and dried on the hot plate. Thereafter, the digests were transferred to a beaker, and adjusted to the volume of 50 ml using deionized water and 1.5 ml CaCl_2_. Atomic absorption spectrophotometry (Perkin Elmer Zeeman 5100 PC) was used to determine Na content. Different spiking of the sodium was then used for the recovery analytic procedure.

### 2.4 Sensory evaluation

Sensory analysis of the samples was accomplished after 2 days of storage by seven trained panelists, as previously described (Berizi et al., 2016). The panelist performed the analysis under white fluorescent lighting to qualify different attributes, such as color, odor, taste, mouthfeel, texture, and total acceptance. The 5‐point scale was employed to score the samples as follows: 5 = excellent, 4 = good, 3 = acceptable, 2 = fair, and 1 = unacceptable.

### 2.5 Texture profile analysis (TPA)

Textural properties of the specimens were studied after 2 days of storage using a texture analyzer (CT3 Texture Analyzer, Brookfield, USA) with a load cell of 30 kg. Sausage samples were removed from the casing, cut into cubes (2.5 cm × 2.5 cm × 2.5 cm), and compressed to 20% of their original heights by two consecutive compressions using a cylindrical probe of 100 mm diameter. Pretest speed, test speed, and post‐test speed were reported as 5, 2, and 10 mm/s, respectively. The timing interval between the two cycles of the TPA test was 10 s, and trigger force was 5 g. Parameters such as hardness, energy required for compressing each specimen, cohesiveness, springiness, gumminess, and chewiness were assessed according to Bourne (1978). All textural parameters were measured at 22 ± 2°C on three replicates (Bourne, 1978).

### 2.6 Microbiological analysis

Two samples from each treatment were used for microbial analysis on days 0, 7, 14, 21, and 28. To prepare a 1:10 dilution, 25 g of each sample was added to 225 ml of sterile 0.1% peptone water (Oxoid, UK), and properly mixed for 1 min and 20 s in a stomacher machine (AES Laboratoire). The pour plate method was then used for microbiological analysis, which was performed according to the instructions by the American Pharmacists Association (APHA) (Speck, 1984) as follows: (a) The plate count agar (PCA; Merck) was employed to determine total aerobic bacteria counts (TABC) incubated with a double layer at 30°C for 48–72 hr and (b) lactic acid bacteria (LAB) on the MRS agar (Merck) incubated with a double layer at 30°C for 48 hr.

### 2.7 Thiobarbituric acid (TBA) values

TBA value was studied on days 7, 14, 21, and 28 of refrigeration, as described by Tarladgis et al. (1960). In brief, cooked sausage (1 g) was blended with 10 ml distilled water and washed with filter paper. Then, three solutions were prepared for the subsequent test steps as follows: (a) solution containing 100 ml HCl 0.25 M (Merck), (b) mixture containing TBA (0.187 g) and TCA (7.5 g), and (c) solution containing BHT (1 g) and 50 ml ethanol (96%). First, 1.5 ml of solution (c) was added to (b) and then made up to volume 100 ml with (a) solution. Eventually, 4 ml of reconstituted solution was mixed with 2 ml of filtered sample and then vortexed for 2 min. In the next step, the tubes were capped and heated in a water bath (95°C) for 15 min. When the tubes were cooled at room temperature, they were centrifuged at 1,000 rcf for 10 min at 25°C (Sigma 3–18 K, Sigma Laboratory Centrifuge). UV‐VIS Double Beam Spectrophotometer (HALO DB‐30, Dynamica, UK) was employed to record the absorbance at 532 nm. The TBA values were measured as mg malondialdehyde (MDA)/kg of meat.

### 2.8 Water holding capacity (WHC)

WHC of the sausage batters was calculated according to the methodology described by Wang and Zayas (1992). Each sample (0.3 g) was placed on filter paper (Whatman No. 2, stored over saturated KC1) between two plexiglass plates and pressed for 20 min by a 1 kg weight. Compensating polar planimeter was used to measure WHC. The following equation was finally used to calculate WHC:

WHC =1‐(total area‐meat film area/meat film area)

### 2.9 Statistical analysis

One‐way ANOVA was used independently for each experiment (SPSS 17, 2002; SPSS Inc.), data were analyzed in triplicate, and two measurements were performed for each parameter. Duncan's post hoc test was then used to determine the differences between the treatment groups (*p* <.05). Furthermore, statistical analysis of storage time on each property was analyzed using the repeated measurement test (Table 2).

**TABLE 2:** Physical and chemical characteristics of the emulsion‐type sausages with reduced sodium content

	Days	Treatments						
		Control	T1	T2	T3	T4	T5	T6
pH	0	6.16 ± 0.02^a^	6.16 ± 0.02^a^	6.15 ± 0.00^a^	6.15 ± 0.00^a^	6.15 ± 0.00^a^	6.14 ± 0.00^a^	6.15 ± 0.00^a^
	7	6.17 ± 0.00^a^	6.17 ± 0.00^a^	6.17 ± 0.01^a^	6.15 ± 0.00^a^	6.16 ± 0.00^a^	6.16 ± 0.01^a^	6.16 ± 0.00^a^
	14	6.19 ± 0.00^b^	6.19 ± 0.00^b^	6.190±0.00^b^	6.17 ± 0.00^a^	6.17 ± 0.00^a^	6.16 ± 0.00^a^	6.17 ± 0.00^a^
	21	6.16 ± 0.02^c^	6.15 ± 0.00^bc^	6.16 ± 0.01^c^	6.13 ± 0.00^ab^	6.14 ± 0.00^abc^	6.11 ± 0.00^a^	6.14 ± 0.00^bc^
	28	6.13 ± 0.02^a^	6.13 ± 0.02^a^	6.13 ± 0.00^a^	6.10 ± 0.00^a^	6.12 ± 0.00^a^	6.10 ± 0.07^a^	6.12 ± 0.00^a^
TBA (MDA/kg)	7	0.15 ± 0.00^a^	0.15 ± 0.01^a^	0.16 ± 0.01^a^	0.15 ± 0.00^a^	0.16 ± 0.00^a^	0.16 ± 0.00^a^	0.15 ± 0.00^a^
	14	0.22 ± 0.00^a^	0.23 ± 0.02^a^	0.23 ± 0.00^a^	0.23 ± 0.00^a^	0.24 ± 0.00^a^	0.23 ± 0.00^a^	0.24 ± 0.00^a^
	21	0.24 ± 0.00^a^	0.24 ± 0.00^a^	0.23 ± 0.00^a^	0.23 ± 0.00^a^	0.23 ± 0.00^a^	0.23 ± 0.00^a^	0.23 ± 0.00^a^
	28	0.24 ± 0.00^a^	0.25 ± 0.00^a^	0.26 ± 0.00^a^	0.25 ± 0.00^a^	0.26 ± 0.00^a^	0.26 ± 0.01^a^	0.26 ± 0.00^a^
*a*_w_ (batter)	0	0.96 ± 0.00^a^	0.96 ± 0.00^a^	0.96 ± 0.00^a^	0.95 ± 0.00^a^	0.95 ± 0.00^a^	0.95 ± 0.00^a^	0.95 ± 0.00^a^
*a*_w_ (product)	0	0.98 ± 0.00^a^	0.98 ± 0.00^a^	0.98 ± 0.00^a^	0.98 ± 0.00^a^	0.98 ± 0.00^a^	0.97 ± 0.00^a^	0.98 ± 0.00^a^
WHC (batter)	0	26 ± 10.60^a^	25 ± 9.89^a^	24 ± 16.97^a^	29 ± 2.82^a^	28 ± 2.82^a^	35 ± 4.24^a^	32 ± 2.82^a^
Moisture	0	66.25 ± 0.14^a^	66.33 ± 0.46^a^	66.99 ± 0.69^a^	66.15 ± 0.19^a^	65.44 ± 0.59^a^	65.01 ± 0.01^a^	65.35 ± 1.15^a^
Na (mg/100 g)	28	683.20 ± 21.35^d^	539.15 ± 9.26^b^	486.55 ± 14.49^a^	552.60 ± 33.51^bc^	510.90 ± 20.64^ab^	592.80 ± 25.73^c^	528.75 ± 10.53^ab^

Note

Averages followed by the same letter, the same line, and the same day did not present significant difference (*p *˂ .05) by test. Control: 1.5% NaCl; T1: 1.2% NaCl + 0.3% KCl (20% of NaCl reduction); T2: 0.9% NaCl + 0.6% KCl (40% of NaCl reduction); T3: 1.2% NaCl + 0.3% KCl + 1% of commercial YE (20% of NaCl reduction); T4: 0.9% NaCl + 0.6% KCl + 1% commercial YE (40% of NaCl reduction); T5: 1.2% NaCl + 0.3% KCl + 2% of commercial YE (20% of NaCl reduction); and T6: 0.9% NaCl + 0.6% KCl + 2% commercial YE (40% of NaCl reduction).

## 3 RESULTS

### 3.1 Physicochemical characteristics

Our results indicated that salt reduction had no effect on pH changes, and pH decreased significantly over time in all the experimental and control groups (*p* <.05) (Table 2). The *a*_w_ values were not significantly changed both in the batter and cooked sausage products (*p* >.05) (Table 2). The sodium content decreased from 683.2 mg/100 g in the control group to 486.55 mg/100 g in the reduced sodium sausages. Not surprisingly, the replacement of NaCl by KCl significantly reduced the sodium content of the sausages (*p* <.05). Moreover, the moisture content was not statistically significant between the control and different treatments (*p* >.05) (Table 2).

### 3.2 Water holding capacity (WHC)

In sausage batter, the values of WHC were numerically higher in the 1.2% NaCl, 0.3% KCl, and 2% YE‐treated group compared to control and other experimental groups (*p* >.05) (Table 2).

### 3.3 Thiobarbituric acid (TBA) values

During storage, the MDA content in the control and treatment groups was significantly enhanced (*p* <.05) (Table 2). However, no substantial changes were detected in the amount of MDA between the control and treatment groups through the cold storage (*p* >.05).

### 3.4 Microbiological characteristics

The details of the microbiological properties of the reduced NaCl content of cooked sausages are given in Table 3. The growth of LAB started on day 14 and continued until day 28. In addition, the LAB numbers were not significantly changed in any of the days among different experimental groups (*p* >.05). Concerning the TABC during the storage of sausages, an increase was observed between day 0 and day 28 of approximately 3 log cycles (Table 3). There was no significant difference in the TABC in all the treatment and control groups (*p* >.05).

**TABLE 3:** Microbiological characteristics (Log CFU/g) of the production of emulsion‐type sausages with reduced sodium content

	Day	Treatments						
		Control	T1	T2	T3	T4	T5	T6
LAB	0	<1/(10^–1^ × 1)	<1/(10^–1^ × 1)	<1/(10^–1^ × 1)	<1/(10^–1^ × 1)	<1/(10^–1^ × 1)	<1/(10^–1^ × 1)	<1/(10^–1^ × 1)
	7	<1/(10–1 × 1	<1/(10^–1^ × 1)	<1/(10^–1^ × 1)	<1/(10^–1^ × 1)	<1/(10^–1^ × 1)	<1/(10^–1^ × 1)	<1/(10^–1^ × 1)
	14	0.50 ± 0.70^a^	1.55 ± 0.07^a^	1.44 ± 0.05^a^	1.00 ± 0.42^a^	1.47 ± 0.24^a^	1.30 ± 0.42^a^	0.35 ± 0.49^a^
	21	0.65 ± 0.91^a^	1.80 ± 0.00^a^	1.65 ± 0.06^a^	1.23 ± 0.33^a^	1.82 ± 0.18^a^	1.57 ± 0.38^a^	0.50 ± 0.70^a^
	28	1.54 ± 0.33^a^	1.90 ± 0.14^a^	1.83 ± 0.07^a^	1.45 ± 0.21^a^	1.87 ± 0.03^a^	2.01 ± 0.09^a^	1.54 ± 0.08^a^
TABC	0	<1/(10^–1^ × 1)^a^	1.47 ± 0.00^c^	1.15 ± 0.21^bc^	1.23 ± 0.33^bc^	0.50 ± 0.70^ab^	<1/(10^–1^ × 1)^a^	<1/(10^–1^ × 1)^a^
	7	1.84 ± 0.08^a^	2.00 ± 0.14^a^	1.84 ± 0.33^a^	2.02 ± 0.25^a^	1.95 ± 0.06^a^	2.16 ± 0.45^a^	2.17 ± 0.56^a^
	14	2.63 ± 0.08^b^	2.94 ± 0.05^c^	2.27 ± 0.03^a^	3.14 ± 0.04^d^	2.91 ± 0.04^c^	2.57 ± 0.03^b^	3.16 ± 0.09^d^
	21	2.99 ± 0.06^a^	3.23 ± 0.12^a^	2.47 ± 0.66^a^	3.22 ± 0.03^a^	3.02 ± 0.02^a^	2.60 ± 0.61^a^	3.23 ± 0.12^a^
	28	3.20 ± 0.14^a^	3.24 ± 0.05^a^	3.06 ± 0.01^a^	3.31 ± 0.01^a^	3.15 ± 0.06^a^	3.13 ± 0.01^a^	3.26 ± 0.76^a^

Note

Averages followed by the same letter, the same line, and the same day did not present significant difference (*p *˂ 0.05) by test. Control: 1.5% NaCl; T1: 1.2% NaCl + 0.3% KCl (20% of NaCl reduction); T2: 0.9% NaCl + 0.6% KCl (40% of NaCl reduction); T3: 1.2% NaCl + 0.3% KCl + 1% of commercial YE (20% of NaCl reduction); T4: 0.9% NaCl + 0.6% KCl + 1% commercial YE (40% of NaCl reduction); T5: 1.2% NaCl + 0.3% KCl + 2% of commercial YE (20% of NaCl reduction); and T6: 0.9% NaCl + 0.6% KCl + 2% commercial YE (40% of NaCl reduction).

### 3.5 Sensory properties

The results of sensory properties for both control and experimental groups are summarized in Table 4. In general, the scores of different parameters assigned by panelists were similar in all treatments. No significant differences were found between the scores for taste in the control and all treatment groups (*p* >.05). Furthermore, no significant alterations in the color, odor, mouthfeel, texture, and total acceptance were observed between the control and treatment samples (*p* >.05).

**TABLE 4:** Sensory evaluation of the production of emulsion‐type sausages with reduced sodium content

Treatments	Color	Odor	Taste	Mouthfeel	Texture	Total acceptance
Control	3.11 ± 1.05^a^	3.33 ± 1.22^a^	3.11 ± 1.05^a^	3.11 ± 0.92^a^	3.22 ± 0.97^a^	3.27 ± 1.25^a^
T1	3.00 ± 1.00^a^	3.00 ± 1.32^a^	2.22 ± 1.20^a^	2.66 ± 0.86^a^	3.44 ± 0.72^a^	2.94 ± 0.88^a^
T2	2.88 ± 1.36^a^	2.88 ± 1.26^a^	2.88 ± 1.05^a^	2.55 ± 1.01^a^	3.22 ± 0.83^a^	3.05 ± 1.07^a^
T3	3.11 ± 1.05^a^	3.44 ± 1.01^a^	3.44 ± 0.88^a^	3.44 ± 1.01^a^	3.44 ± 0.88^a^	3.33 ± 0.70^a^
T4	3.11 ± 1.16^a^	3.11 ± 1.05^a^	2.77 ± 1.20^a^	2.88 ± 1.05^a^	3.44 ± 1.01^a^	3.38 ± 1.05^a^
T5	3.11 ± 1.16^a^	2.88 ± 1.05^a^	2.88 ± 1.16^a^	2.77 ± 0.83^a^	3.22 ± 0.83^a^	3.27 ± 0.90^a^
T6	3.11 ± 1.16^a^	3.77 ± 0.97^a^	3.55 ± 0.72^a^	3.55 ± 0.52^a^	3.44 ± 0.88^a^	3.72 ± 0.56^a^

Note

Averages followed by the same letter, the same line, and the same day did not present significant difference (*p *˂ .05) by test. Control: 1.5% NaCl; T1: 1.2% NaCl + 0.3% KCl (20% of NaCl reduction); T2: 0.9% NaCl + 0.6% KCl (40% of NaCl reduction); T3: 1.2% NaCl + 0.3% KCl + 1% of commercial YE (20% of NaCl reduction); T4: 0.9% NaCl + 0.6% KCl + 1% commercial YE (40% of NaCl reduction); T5: 1.2% NaCl + 0.3% KCl + 2% of commercial YE (20% of NaCl reduction); and T6: 0.9% NaCl + 0.6% KCl + 2% commercial YE (40% of NaCl reduction).

### 3.6 Textural properties

Textural values of the cooked samples containing reduced NaCl are given in Table 5. In terms of maximum force, no significant difference was found between the samples. In the current study, we did not witness any significant impact regarding the parameters of springiness, gumminess, hardness, cohesiveness, and chewiness (*p* >.05).

**TABLE 5:** Parameters from instrumental texture profile analysis in emulsion‐type sausages with reduced sodium content

	Treatments						
	Control	T1	T2	T3	T4	T5	T6
Hardness (g)	27.59 ± 1.84^a^	28.68 ± 2.23^a^	26.43 ± 1.61^a^	27.69 ± 1.16^a^	28.51 ± 3.44^a^	27.25 ± 1.31^a^	25.91 ± 1.14^a^
Cohesiveness (ratio)	0.53 ± 0.01^a^	0.56 ± 0.05^a^	0.52 ± 0.03^a^	0.56 ± 0.02^a^	0.55 ± 0.05^a^	0.53 ± 0.06^a^	0.51 ± 0.04^a^
Springiness (cm)	1.21 ± 0.01^a^	1.21 ± 0.01^a^	1.20 ± 0.00^a^	1.22 ± 0.00^a^	1.21 ± 0.01^a^	1.21 ± 0.01^a^	1.20 ± 0.00^a^
Gumminess (*N*/cm^2^)	14.58 ± 0.81^a^	16.07 ± 2.51^a^	13.76 ± 1.66^a^	15.43 ± 0.71^a^	15.58 ± 2.45^a^	14.31 ± 1.46^a^	13.23 ± 1.29^a^
Chewiness (*N*/cm)	0.17 ± 0.00^a^	0.19 ± 0.03^a^	0.16 ± 0.02^a^	0.18 ± 0.00^a^	0.18 ± 0.02^a^	0.17 ± 0.01^a^	0.15 ± 0.01^a^

Note

Averages followed by the same letter, the same line, and the same day did not present significant difference (*p *˂ .05) by test. Control: 1.5% NaCl; T1: 1.2% NaCl + 0.3% KCl (20% of NaCl reduction); T2: 0.9% NaCl + 0.6% KCl (40% of NaCl reduction); T3: 1.2% NaCl + 0.3% KCl + 1% of commercial YE (20% of NaCl reduction); T4: 0.9% NaCl + 0.6% KCl + 1% commercial YE (40% of NaCl reduction); T5: 1.2% NaCl + 0.3% KCl + 2% of commercial YE (20% of NaCl reduction); and T6: 0.9% NaCl + 0.6% KCl + 2% commercial YE (40% of NaCl reduction).

## 4 DISCUSSION

The type of meat and its pH play an important role in meat products. Generally, using fresh meat is the best choice for these products because of its high solubility in proteins, but manufacturing companies have to use frozen meat (with a relatively lower pH) because of the shortage and unavailability of this volume of fresh meat. An elevation in the pH values was detected in the cooked sausages up to day 14 of production, which happens through the accumulation of base compounds such as ammonia and total volatile basic nitrogen (TVB‐N) resulted from the enzymatic activities of proteolytic bacteria (Ghollasi et al., 2017). Then, the pH values diminished in sausages after day 14 to the end of storage, which was possibly due to the metabolism of carbohydrates to organic acids by LAB (Fraqueza et al., 2008; Özer et al., 2012); meanwhile, the replacement of NaCl by KCl did not influence the pH values (Campagnol et al., 2011). Nevertheless, a slight reduction of the pH values occurred following the addition of YE at the concentrations of 1% to 2% (Table 2); however, this decrease was not substantial and was likely resulted from the composition of sugar in the YE which could be feasibly metabolized by LAB (Campagnol et al., 2011; Laranjo et al., 2016). At the end of cold storage, the pH of products was within the range of 5.8 to 6.2, and this pH is desirable for microbial stability and sensory properties. The pH values of products were not changed by the salt contents. A reduction in salt from 1.5% to 0.5% was previously shown in ham products, while pH was not changed (Lee & Chin, 2011); in addition, in Frankfurter sausages, the amounts of salt did not decrease from 2.5% to 1.0% (Sofos, 1983).

A_w_ values were measured in the batter and cooked sausages in the control and all treatment groups (Table 2). No considerable changes were observed between the experimental and control groups. The results indicated that NaCl replacement with KCl did not affect the water activity values. In this regard, similar findings have been reported by Horita et al. (2014) and Vidal et al., (2020).

Salt increases the water binding of meat, while with reducing salt, the product is expected to lose weight (Ruusunen & Puolanne, 2005). A similar study conducted on fermented sausages revealed that the meat weight decreased from 38.42% to 41.42%. In our study, contrary to other studies, while WHC of T1 and T2 decreased, T3, T4, T5, and T6 increased compared to the control group. The increase of WHC is likely resulted from adding YE; moreover, a slight reduction in the meat weight occurred, which was not statistically significant.

The control sample contained 683.2 mg/100 g of sodium (Table 2). Not surprisingly, the replacement of NaCl significantly reduced the sodium content in all treatments. In treatments where 20% of NaCl was reduced (T1, T3, T5), sodium levels diminished by 21.09%, 19.12%, and 13.24% compared to the control group, respectively. In the groups containing 40% lower NaCl contents (T2, T4, and T6), the values of sodium were reduced to 28.79%, 25.22%, and 22.61%, respectively. The lesser reduction in sodium contents of the YE‐supplemented groups (T3 to T6) was probably due to the presence of tiny amount of sodium in YE.

The TABC and the growth of LAB in all treatments were similar to those in the control group (Table 3). In addition, no significant difference in the TABC and LAB was observed between the control group and different treatments with various salt percentages (Aaslyng et al., 2014; Sofos, 1983). Nevertheless, there was a significant difference in TABC (and not LAB) between days 0 and 14. In this study, using NaCl with KCl did not have any effect on the growth of aerobic microorganisms. These bacteria continuously increased from day 0 up to day 28; a similar finding was reported by Aaslyng et al. (2014), where the pH value increased until day 14.

The LAB developed quickly from days 14 to 28 after the production (Aaslyng et al., 2014; Andres et al., 2006), and with the growth and proliferation of LAB, the pH declined (Table 2). In the groups which used YE, no significant difference was observed in sausages in either the TABC or LAB between different treatments and control groups.

The results of sensory evaluation (Table 4) revealed that the replacement of 20% and 40% salts did not change the color, texture, odor, taste, mouthfeel, and total acceptance attributes compared to the controls. On the other hand, an improvement of the odor, taste, mouthfeel, and total acceptance was investigated after supplementing of 2% YE and 40% NaCl reduction (T6). This finding was basically resulted from an increase in the volatile components present in the YE, as described earlier (Campagnol et al., 2011).

In emulsified meat products, such as cooked emulsion sausages, the texture is one of the most important sensory properties. When the NaCl level is reduced, the solubility of myofibrillar proteins and ionic strength also decrease, thereby lowering the water holding capacity and gel strength (Gordon & Barbut, 1992; Whiting, 1984). In this study, concerning the texture properties, such as springiness, gumminess, hardness, cohesiveness, and chewiness, with the replacement of 20% and 40% NaCl by KCl, no significant difference was detected between the control and treatment groups. In addition, using KCl as a substitute for NaCl in a suitable concentration can compensate for salt loss. In this study, contrary to the results reported by Horita et al. (2014), reduction of salt content did not show any increase in hardness. WHC is very important in improving the quality of the texture and product, where the type of meat consumed, and the type and amount of additives authorized in the batter WHC have a major role (Dzudie et al., 2002; Sharifian & Attaran, 2014). Neutralized salts increase their protein solubility at low concentrations, which is due to the presence of salt ions among the protein chains, and to prevent the bonding between them. The excessive salt leads to diminished solubility of myofibril proteins. The reason for decreased solubility in this case is the absorption of water by a large number of salt ions limiting protein access to water molecules (Paula et al., 2019). In a study by Ruusunen and Puolanne (2005), NaCl reduction and lowering pH proved to reduce the water holding capacity; however, in the present study, with reducing the amount of NaCl and adding KCl, the WHC decreased compared to the control group, indicating the lower potency of potassium chloride. However, in the treatment groups with reduced amounts of salt and added amounts of YE, a rise was seen in WHC, possibly due to the presence of minor sodium and its low moisture content, and the increase of WHC may be due to the addition of YE.

The oxidation of fat in food is due to the existence of different types of reactions leading to the formation of free radicals, hydroperoxides, and other products of corruption (Domínguez et al., 2014). Meat products decay in response to the interaction of various types of radicals or through fat oxidation (Fernández et al., 1997). The amount of fat in meat has a direct relationship with the extent of fat oxidation, where poultry meat has a better quality because of its lower fat content compared to other meats. This was in line with the findings of Bhattacharyya et al. (2007). The TBA values indicated a significant ascending trial during the storage period; however, these values did not exceed the predictable off‐odors or off‐flavor, to indicate the rancidity (Greene & Cumuze, 1982).

Changes in the sensory evaluation (including color, taste, odor, mouth feeling, texture, and total acceptance) were not obvious among different groups. Our results showed that replacement of NaCl by KCl + YE revealed improved sensory evaluation compared to the replacement of NaCl by KCl alone. In the groups using 2% YE (T5 and T6), the best score of sensory evaluations was recorded, which was due to covering metal taste of KCl. Moreover, the production of favorite aromatic compounds by YE was entirely covered the bitter taste of KCl. These results were strongly supported by previous works (Desmond, 2006; Vidal et al., 2020).

## 5 CONCLUSIONS

According to the results of present study, the replacement of 20% to 40% NaCl in emulsion‐type sausages with KCl did not have adverse effects on textural, microbiological, and sensory properties, and it did not result in any significant changes in the overall acceptable pleasantness. Emulsion‐type sausages treated with YE had a better WHC than other treatments. The increased values of taste and odor were associated with the presence of YE because it resolved the defects in the sensory qualities due to using the KCl. Thus, producing harmless emulsion‐type sausages with an overall acceptability is industrially practical.

## CONFLICT OF INTERESTS

The author(s) declares no conflict of interests.

## AUTHORS’ CONTRIBUTIONS

Enayat Berizi and Seyed Shahram Shekarforoush: study design; analysis and interpretation of data; drafting and finalizing the manuscript; and study supervision. Majid Mohammadzadeh: carried out the experimental works; data analyses; and drafted the first version of the manuscript.

## ETHICAL APPROVAL

In this research, human participants or animal studies were not necessary. The manuscript is not currently being considered for publication in any other journal.
